# Personal

**Published:** 1917-10

**Authors:** 


### PERSONAL

Dr. Edgar A. Forsyth announces the removal of his office to 471 Virginia St.; Tupper 157.

---

Dr. E. M. Manwaren of 232 Ferry St., Buffalo, returned to Buffalo Sept. 5, after an extended visit to relatives in Oswego.

---

The house of Dr. Wm. L. Phillips of Buffalo, was ransacked by burglars during the absence of the family, on the night of Sept. 3.

---

Dr. Herbert A. Smith of Buffalo, Captain, M. R. C., is absent from the city on service.

---

Dr. James J. Clements of Buffalo has been commissioned 1st Lt., M. R. C.

---

Dr. Edward Durney of Buffalo has been appointed Deputy Health Commissioner during the absence of Dr. Arthur C. Schaefer on Military duty with the 74th Reg. Dr. Schaefer is a Captain in the medical corps.

Lts. J. A. Bennett, Elmira; I. W. Livermore, Gowanda, and G. E. Stevenson, Penn Yan, have been ordered to Ft. Ethan Allen, Vt.

---

Dr. Paul Betawski of Bath, Chief Surgeon of the Soldiers' Home, has resigned his position and accepted one as 1st Lt. in the M. R. C. On leaving for the training camp at Ft. Benjamin Harrison, he was accompanied to the train by a large number of veterans, officers and employees of the Home and citizens of Bath.

---

Dr. F. A. Walder of Lockport has been commissioned 1st Lt. M. R. C. and has gone to Ft. Benjamin Harrison.

---

A Company, Tonawanda Home Defense Corps, includes the following physicians: H. S. Wende, Mess Sergeant; J. Albert Hobbie and Herman U. Berger, corporals.

---

Dr. H. R. Gaylord of Buffalo, Major, M. R. C., is stationed at Allentown, Pa. He was in Buffalo the middle of Sept. to direct the physical examination of recruits.

---

The following members of the medical profession of western N. Y. have been ordered to Ft. Benjamin Harrison: Lts. F. H. Mildrew, Auburn; W. L. Weeden, Clifton Springs; R. S. Barry, J. L. Bishop and F. J. Talbot, Niagara Falls; C. A. Grrenleaf, Olean; L. J. Barber, B. H. Dike, P. M. Parker, Rochester; Chas. A. Joy, Sonyea; R. S. Simpson, Lyons.

---

Lt. D. S. Childs of Syracuse has been ordered to Camp Travis, Ft. Sam Houston, Texas, as roentgenologist. Capt. U. S. Kann of Binghamton has been ordered to similar duty at Camp Wadsworth, Spartansburg, S. C.

The following have been ordered to duty at Syracuse: Major E. S. Van Duyn, Capt. Archer D. Babcock and Lt. B. C. Doust of Syracuse.

---

Lt. Edward L. Hanes of Rochester has been ordered to Washington Barracks to make examinations in his specialty.

---

Dr. Joseph Brumberg of Buffalo has been commissioned 1st Lt. in the M. R. C.

---

Dr. Albert Warren Ferris has finished his work as Superintending Director for the State of N. Y. at the Saratoga Springs Reservation and has returned to his position on the medical staff of the Glen Springs, Watkins, N. Y.

